# The Predictive Value of Interleukin-6 and Neutrophil-Lymphocyte Ratio in Patients with Severe and Extremely Severe Oral and Maxillofacial Space Infections

**DOI:** 10.1155/2021/2615059

**Published:** 2021-01-28

**Authors:** Li Xiaojie, Liu Hui, Gong Zhongcheng, Wang Chenggang, Niu Yaqi

**Affiliations:** ^1^Oral and Maxillofacial Surgical Ward Medical Center, The Affiliated Stomatological Hospital of Soochow University, Suzhou Stomatological Hospital, Suzhou 215000, China; ^2^Department of Oral and Maxillofacial Surgery, Shanghai Stomatological Hospital, Fudan University, Shanghai 200001, China; ^3^Oncology Department of Oral and Maxillofacial Surgery, The First Affiliated Hospital of Xinjiang Medical University, Urumqi 830054, China

## Abstract

**Objective:**

To investigate the correlation between clinical manifestation and neutrophil-lymphocyte ratio, C-reactive protein (CRP), and interleukin-6 (IL-6) in the patients with severe and extremely severe oral and maxillofacial space infection (OMSI).

**Methods:**

In this retrospective study, we included 18 patients with severe and extremely severe OMSI from November 2012 to October 2018. Pearson or Spearman correlation coefficients were calculated to measure the association between the number of complications and locations and the number and percentage of lymphocyte, leukocyte, neutrophil, eosinophil, basophil, monocyte, CRP, and IL-6. A multivariable regression model was used to predict the number of complications and locations from these measures.

**Results:**

IL-6 was positively correlated with neutrophil-lymphocyte ratio (*r*_s_ = 0.773, *P* = 0.005), percentage of neutrophil (*r*_s_ = 0.927, *P* = 0.001), and the number of neutrophil (*r*_s_ = 0.627, *P* = 0.039). It was negatively correlated with percentage of monocyte (*r*_s_ = −0.773, *P* = 0.005). CRP was positively correlated with neutrophil-lymphocyte ratio (*r*_s_ = 0.556, *P* = 0.020) and percentage of neutrophil (*r*_s_ = 0.515, *P* = 0.035). It was negatively correlated with the number of lymphocyte (*r*_s_ = −0.517, *P* = 0.017), percentage of lymphocyte (*r*_s_ = −0.578, *P* = 0.015), number of eosinophil (*r*_s_ = −0.560, *P* = 0.020), percentage of eosinophil (*r*_s_ = −0.504, *P* = 0.039), number of basophil (*r*_s_ = −0.504, *P* = 0.039), and percentage of basophil (*r*_s_ = −0.548, *P* = 0.023). The number of the involved organs was positively correlated with neutrophil-lymphocyte ratio (*r*_s_ = 0.511, *P* = 0.030). The number of complications was positively correlated with the percentage of neutrophils (*r* = 0.738, *P* = 0.001), the neutrophil-lymphocyte ratio (*r* = 0.576, *P* = 0.012), IL-6 (*r*_s_ = 0.907, *P* = 0.001), and CRP (*r*_s_ = 0.599, *P* = 0.011). Multivariable regression analysis showed that the neutrophil-lymphocyte ratio (*β* = 0.080) was a significant predictor of the number of the involved organs and the neutrophil-lymphocyte ratio (*β* = 0.099). In addition, IL-6 (*β* = 0.002) was a significant predictor of the number of complications.

**Conclusions:**

The neutrophil-lymphocyte ratio and IL-6 contributed to the assessment of general condition in severe and profound OMSI patients. These parameters can be used as predictors to evaluate the severity of OMSI.

## 1. Introduction

Oral and maxillofacial space infection (OMSI) usually occurs in the space between the oral, facial, and deep cervical parts, which is potentially associated with the infection in the loose connective tissues or the adipose tissues. According to the involved sites, scale, and the presence of airway obstruction, OMSI is classified into mild, moderate, severe, and extremely severe types. Generally, patients with severe OMSI present involvement in the parapharyngeal space, posterior pharyngeal space, and anterior tracheal space, while those with extremely severe OMSI presented mediastinal and intracranial infection. Severe OMSI is one of the types of deep cervical infection, which commonly complicates with acute respiratory obstruction, mediastinal inflammation, Lemierre's syndrome, suppurative thrombophlebitis of the internal jugular vein, cervical necrotizing fasciitis, and pneumonia [1]. Particularly, in the presence of patients with extremely severe OMSI, many would present poor prognosis due to descending necrotizing mediastinitis, which resulted in a mortality of up to 40% [2].

A large number of OMSI patients show host responses and humoral immunity, such as activation of complements, acute-phase proteins, cytokines, monocytes, macrophages, and anti-inflammatory mediators. Neutrophil plays an important role in the process of infectious disease [3]. Neutrophil and lymphocyte play an essential role in process of infection among the leukocyte subpopulation. In the process of infection, increasing neutrophil is a typical response of leukocyte [4]. Neutrophil-to-lymphocyte ratio (NLR) may more sensitive to indicate the extension of OMSI than other WBC subpopulations [5]. As an acute-phase protein, CRP is a rapidly elevated protein in the presence of infection, inflammation, and trauma [6]. As a proinflammatory cytokine, IL-6 was available in peripheral blood at the early stage of the infection, which then led to the generation of CRP in the liver. At the early stage, IL-6 level was higher than CRP level, which can be helpful to the early diagnosis [7]. To date, few studies focused on the prediction of the severe and extremely severe infections based on CRP, IL-6, or NRL. In this study, we aimed to investigate the correlation between these factors and the OMSI complications.

## 2. Materials and Methods

### 2.1. Patients

In this retrospective study, we included 18 patients with severe and extremely severe OMSI from November 2012 to October 2018. Those with oral and maxillofacial space infection were included in this study. The exclusion criteria were as follows: patients with a history of antibiotic treatment of >10 days prior to admission, immunological diseases, abnormal liver and kidney functions, cancer patients, patients with alcohol or drug addiction, pregnant women, those with a BMI of >30, and those received hormones during treatment. Each patient signed the informed consent. The study protocols were approved by the Ethical Committee of the First Affiliated Hospital of Xinjiang Medical University.

### 2.2. Laboratory Examination

The routine full and differential blood cell counts, CRP, and IL-6 of patients were tested within 2 h after admission. The blood samples were collected using tubes containing 5 ml anticoagulated venous blood (EDTA). The tube was centrifugated at 3,000 rpm for 10 min. The tested serological parameters included lymphocyte, leukocyte, neutrophil, eosinophil, basophil, monocyte (Beckman DxH 600 automated blood analyzer, USA), IL-6 (Liuyi WD-2102A, China), and C-reactive protein (Beckman AU5800, USA).

### 2.3. Statistical Analysis

Data analysis was conducted using the SPSS 20.0 software. The Shapiro-Wilk method was utilized to verify the normality of the data. Data that were normally distributed were presented as the mean ± standard deviation. Data that were not normally distributed were presented as the median (P25, P75). Pearson correlation analysis was given to the dual variables that were normally distributed, and the Spearman correlation analysis was given to these that were not normally distributed. For missing values, listwise deletion was applied, and stepwise method was used for the multiple linear regression analysis. *P* < 0.05 was considered to be statistically significant.

## 3. Results

### 3.1. Patients' Characteristics

In total, 18 patients (male, 12; female, 6) diagnosed with severe and extremely severe OMSI were included in this study. They were in an age range of 16-75 years (M, [P25, P75] = 54, [52, 59]). Based on the disease severity, there were 12 patients with severe OMSI and 6 patients with extremely severe OMSI. For the source of OMSI, 11 cases were of odontogenic infection and 7 cases of adenogenic infection (Table 1).

The involved organs of each patient were 4-11 sites (M, [P25, P75] = 5, [4, 7]). The infection sites included back (*n* = 1), buccal space (*n* = 1), submandibular space (*n* = 16), submental space (*n* = 10), sublingual space (*n* = 3), pterygomaxillary space (*n* = 10), masseter space (*n* = 7), temporal deep space (*n* = 2), temporomandibular space (*n* = 3), parapharyngeal space (*n* = 15), posterior pharyngeal space (*n* = 5), prevertebral space (*n* = 3), neck (*n* = 11), supraclavicular fossa (*n* = 6), pretracheal space (*n* = 3), anterior esophagus (*n* = 1), and mediastinum (*n* = 6). Seventeen showed complications. The number of complications per patient was 0-7 (mean ± SD = 3.44, 2.33). In total, 17 patients had hypoproteinemia, 7 patients had liver dysfunction, 6 patients had pneumonia, 6 patients had pleural effusion, 4 patients had respiratory obstruction, 4 patients had atelectasis, 4 patients had pericardial effusion, 4 patients had renal insufficiency, 2 patients had anemia, 2 patients had esophageal fistula, 2 patients had tracheal fistula, 1 patient had pulmonary embolism, 1 patient had bacteremia, 1 patient had sepsis, and 1 patient had septic shock (Table 1).

### 3.2. Clinic and Laboratory Examination Findings

The length of hospital stay was in a range of 5-54 days (M, [P25, P75] = 20, [13, 26]). The number of lymphocyte was 0.33 − 3.26 × 10^9^/l (1.46 ± 0.94). The percentage of lymphocyte was 2.2%-43% (M, [P25, P75] = 10.22, [4.6, 15.2]). The neutrophil-lymphocyte ratio was 3.62%-41.86% (M, [P25, P75] = 9.53, [5.14, 18.68]). The number of leukocyte was 8.68 − 26.49 × 10^9^/l(M, [P25, P75] = 12.92, [11.36, 15.60]). The percentage of neutrophils was 72.8%-95.3% (83.74 ± 6.94) at admission. The number of neutrophils was 6.35 − 21.32 × 10^9^/l (12.04 ± 4.17). The number of eosinophil was 0 − 0.13 × 10^9^/l(M, [P25, P75] = 0.005, [0, 0.02]). The percentage of eosinophil was 0-1.7% (M, [P25, P75] = 0.07, [0, 0.1]). The number of basophil was 0 − 0.14 × 10^9^/l(M, [P25, P75] = 0.02, [0.01, 0.03]). The percentage of basophil was 0-0.7% (M, [P25, P75] = 0.2, [0.1, 0.3]). The number of monocyte was 0.18 − 1.99 × 10^9^/l (0.79 ± 0.49). The percentage of monocyte was 1.4-11.1% (5.58 ± 2.96). Except for 7 patients did not undergo the IL-6 test, the results of IL-6 of the remaining 11 patients were 7.67-2,193.00 pg/ml (M, [P25, P75] = 87.76, [31.75, 388.45]). One patient denied the CPR determination upon admission; the CPR of the remaining 17 patients was 4.43-279.66 mg/l (M, [P25, P75] = 19.52, [11.45, 78.28]) (Table 2).

### 3.3. Correlation between the IL-6 and CRP with Blood Values

As shown in Table 3, IL-6 was positively correlated with neutrophil-lymphocyte ratio (*r*_s_ = 0.773, *P* = 0.005), percentage of neutrophil (*r*_s_ = 0.927, *P* = 0.001), and number of neutrophil (*r*_s_ = 0.627, *P* = 0.039). It was negatively correlated with percentage of monocyte (*r*_s_ = −0.773, *P* = 0.005). CRP was positively correlated with neutrophil-lymphocyte ratio (*r*_s_ = 0.556, *P* = 0.020) and percentage of neutrophil (*r*_s_ = 0.515, *P* = 0.035). It was negatively correlated with number of lymphocyte (*r*_s_ = −0.517, *P* = 0.017), percentage of lymphocyte (*r*_s_ = −0.578, *P* = 0.015), number of eosinophil (*r*_s_ = −0.560, *P* = 0.020), percentage of eosinophil (*r*_s_ = −0.504, *P* = 0.039), number of basophil (*r*_s_ = −0.504, *P* = 0.039), and percentage of basophil (*r*_s_ = −0.548, *P* = 0.023) (Table 3).

### 3.4. Correlation between Neutrophil-Lymphocyte Ratio and the Number of the Involved Organs

**Figure 1:**
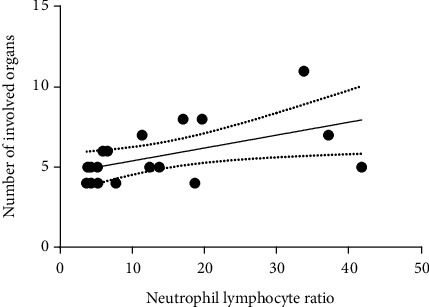
The positive correlation between neutrophil-lymphocyte ratio (*r*_s_ = 0.511, *P* = 0.030) and number of the involved organs.

As shown in Figure 1, the number of the involved organs showed a positive correlation with neutrophil-lymphocyte ratio (*r* = 0.511, *P* = 0.030). There were no differences with statistical significance for other WBC subpopulations, IL-6 and CRP (Table 4).

### 3.5. Correlation between the Percentage of Neutrophils, Neutrophil-Lymphocyte Ratio, IL-6, CRP, and the Number of Complications

The number of complications was positively correlated with the percentage of neutrophils (*r* = 0.738, *P* = 0.001), the neutrophil-lymphocyte ratio (*r* = 0.576, *P* = 0.012), IL-6 (*r*_s_ = 0.907, *P* = 0.001), and CRP (*r*_s_ = 0.599, *P* = 0.011). Other WBC subpoplulations were not statistically significant (Table 5).

### 3.6. Multiple Linear Regression Analysis of the Number of the Involved Organs

In this section, using the percentage of neutrophils and neutrophil-lymphocyte ratio as independent variables and the number of the involved organs as dependent variables in multiple linear regression analysis, stepwise regression was carried out in one step. Adjusted coefficient of determination (*R*_adj_^2^) of the models was 0.225. Therefore, it was the optimal equation (*F* = 5.949, *P* = 0.027). The fitted regression equation was statistically significant. Importantly, neutrophil-lymphocyte ratio was found to be a statistically significant predictor of the number of the involved organs (Table 6). However, the mean of studentized residual was -2.256 to 2.520 and Cook's distance was 0.001 to 1.467 with 18 objectives.

### 3.7. Multiple Linear Regression Analysis of the Number of Complications

In this section, using the percentage of neutrophils, neutrophil-lymphocyte ratio, IL-6, and CRP as independent variables as well as the number of complications as dependent variables in multiple linear regression analysis, stepwise regression was carried out in 4 steps. Adjusted coefficient of determination (*R*_adj_^2^) of the two models was 0.904. Therefore, it was the optimal equation (*F* = 47.912, *P* = 0.001). The fitted regression equation was statistically significant. Importantly, the neutrophil-lymphocyte ratio and IL-6 were significant predictors for the number of complications (Table 7). However, the mean of studentized residual was -1.261 to 1.561 and Cook's distance was 0.006 to 10.132 with 11 objectives.

## 4. Discussion

OMSI is a common disease in oral and maxillofacial regions. These patients may show complications or even life-threatening conditions, in the absence of appropriate treatment. To date, extensive studies have been conducted to screen the potential markers in order to distinguish high-risk and low-risk space infections.

Neutrophil-lymphocyte ratio (NLR) was positively correlated with the number of infection sites in patients with severe OMSI. Patients with more infection sites usually showed higher neutrophil-lymphocyte ratio. Neutrophils have been considered one of the main factors involving in the innate immune response, as they present antibacterial and cytotoxic effects, as well as multiple immunomodulatory functions such as proinflammatory and anti-inflammatory effects [4, 8]. These indicated that percentage of neutrophils contributed to the estimation of the infection sites of the OMSI patients.

NLR, as an infectious parameter which is easy and quick to get, is an indicator for imbalance of systemic cell-mediated immunity [9]. It reported that NLR assisted in predicting the prognosis of infectious disease [10–12]. However, NLR was considered to be an indicator for some infectious tumor and cardiovascular diseases [13–15].

In our study, IL-6 showed a higher correlation with neutrophil-lymphocyte ratio (*r*_s_ = 0.773, *P* = 0.005), percentage of neutrophil (*r*_s_ = 0.927, *P* = 0.001), which meant IL-6 might serve as an indicator for evaluating the severity of the OMSI.

In our study, the number of the involved organs showed a positive correlation with neutrophil-lymphocyte ratio. The multiple linear regression analysis showed that a higher NLR was a significant predictor of more involved organs. As the sample size for patients with severe and extremely severe OMSI was small, there might be possibilities of data missing and outliers. Above all, the results should be interpreted rationally, and more cases should be integrated in the subsequent study. In future, more studies are required to investigate the effectiveness of the NLR.

Neutrophils and lymphocytes play essential roles in immune responses [16, 17]. In the presence of physical or physiologic stress, there was increase in the cortisol, neutrophil, and catecholamine levels, as well as decline in the lymphocyte count [17, 18]. Because neutrophil-to-lymphocyte ratio is calculated as neutrophil count divided by lymphocyte count, it can reflect the changes in both types of cells. Therefore, it is considered a more sensitive marker for the evaluation of extent of stress or systemic inflammation than the other cell type alone such as neutrophils and lymphocytes [16]. Our data indicated a positive correlation between the neutrophil-lymphocyte ratio, IL-6, and complications in patients with severe and extremely severe OMSI. In multivariable regression analysis, both neutrophil-lymphocyte ratio and IL-6 entered the equation, and coefficient of determination adjusted coefficients was 0.904. The neutrophil-lymphocyte ratio and IL-6 were the main independent predictors for the number of complications.

Previous studies suggested that complications in OMSI patients were associated with blood glucose, WBC count, systemic disease, neck swelling, and disease duration [8, 19, 20]. The serious complications were related to the pathogenesis of diabetes, multiple space infection, number of WBCs upon admission (≥1.5 × 10^9^/l), age (≥65 years), history of medication, dyspnea, and body temperature (>39°C) [21–23]. Percentage of neutrophils was an independent predictor for serious complications. In addition, there was an association between percentage of neutrophils of >85% and the severity of maxillofacial space infection [16]. Patients with a high percentage of neutrophil-lymphocyte ratio and IL-6 at admission should be alert about the severity of complications in order to control the condition effectively and avoid the occurrence of serious complications. However, in this study, the cook's distance was greater than 0.5 and absolute value of the studentized residuals was greater than 2 from multiple linear regression analysis. On this basis, there might be outliers in these formulas. Neutrophil-lymphocyte ratio and IL-6 should be still tested in further studies, together with illustrating the influence to the complications.

There are some limitations in this study. As the number of cases diagnosed with severe and extremely severe OMSI combined with life-threatening complications is limited, it is difficult to extend to all patients with maxillofacial space infections. The follow-up should expand the scope of application and the sample content. Meanwhile, the blood test indices were not adequate for each patient. In future, more studies involving a large sample size are needed.

In summary, the neutrophil-lymphocyte ratio and IL-6 contributed to assessment of general condition in severe and extremely severe OMSI patients. These parameters could be utilized as predictors to evaluate the severity of OMSI in clinical settings.

## Figures and Tables

**Table 1:** Clinical data of 18 patients with severe or profound OMSI.

No.	Age (yrs)	Gender	Involved organs	Complications
1	52	Male	Submandibular space, submental space, pterygomaxillary space, neck, parapharyngeal space	Hypoproteinemia, respiratory obstruction, pneumonia, pleural effusion, dysfunction, renal insufficiency
2	54	Female	Neck, posterior pharyngeal space, prevertebral space, mediastinum	Pneumonia, hypoproteinemia, esophageal fistula, tracheal fistula, dysfunction, anemia
3	30	Female	Masseter space, pterygomaxillary space, submandibular space, neck, parapharyngeal space	Hypoproteinemia, dysfunction, anemia
4	16	Male	Submandibular space, pterygomaxillary space, parapharyngeal space, neck	None
5	75	Female	Submandibular space, submental space, supraclavicular fossa, parapharyngeal space, pretracheal space	Hypoproteinemia
6	57	Male	Buccal space, masseter space, pterygomaxillary space, submandibular space, submental space, parapharyngeal space	Hypoproteinemia, pneumonia, pleural effusion, atelectasis
7	72	Male	Submandibular space, submental space, sublingual space, parapharyngeal space	Hypoproteinemia, dysfunction
8	56	Male	Infratemporal space, masseter space, pterygomaxillary space, submandibular space, submental space, parapharyngeal space	Hypoproteinemia
9	47	Male	Submandibular space, submental space, supraclavicular fossa, parapharyngeal space, neck	Hypoproteinemia, renal insufficiency
10	67	Female	Pterygomaxillary space, submandibular space, submental space, retropharyngeal space, prevertebral space, parapharyngeal space, pretracheal space, mediastinum	Hypoproteinemia, respiratory obstruction, pleural effusion, pericardial effusion, pulmonary embolism, atelectasis, pneumonia, renal insufficiency
11	57	Male	Masseter space, pterygomaxillary space, submandibular space, supraclavicular fossa, neck, parapharyngeal space, pretracheal space	Respiratory obstruction, hypoproteinemia, renal insufficiency
12	53	Female	Submandibular space, retropharyngeal space, prevertebral space, pretracheal space, mediastinum	Hypoproteinemia
13	42	Female	Submandibular space, supraclavicular fossa, neck, mediastinum	Hypoproteinemia, pleural effusion, bacteremia
14	53	Male	Infratemporal space, masseter space, submandibular space, parapharyngeal space	Hypoproteinemia, respiratory obstruction
15	59	Male	Submandibular space, submental space, sublingual space, back, neck, parapharyngeal space, mediastinum	Pneumonia, atelectasis, tracheal fistula, esophageal fistula, hypoproteinemia, dysfunction
16	52	Male	Pterygomaxillary space, submental space, supraclavicular fossa, neck, parapharyngeal space	Sepsis, septic shock, pericardial effusion, hypoproteinemia, pleural effusion, dysfunction
17	54	Male	Temporal deep space, masseter space, pterygomaxillary space, submandibular space, neck, retropharyngeal space, parapharyngeal space, supraclavicular fossa	Pericardial effusion, hypoproteinemia
18	60	Male	Temporal deep space, infratemporal space, submandibular space, submental space, masseter space, pterygomaxillary space, sublingual space, neck, parapharyngeal space, retropharyngeal space, mediastinum	Pleural effusion, atelectasis, pneumonia, pericardial effusion, hypoproteinemia, dysfunction

**Table 2:** Blood test results and treatment methods of 18 patients with severe or extremely severe OMSI.

No.	Source	Hospital stay (days)	Number of lymphocyte	Percentage of lymphocyte	Neutrophil-lymphocyte ratio	Leukocyte	Percentage of neutrophil	Number of neutrophil	Number of eosinophil	Percentage of eosinophil	Number of basophil	Percentage of basophil	Number of monocyte	Percentage of monocyte	IL-6 (pg/ml)	CPR (mg/l)
1	Adenogenic	23	1.19	10.54	13.73	18.39	88.84	16.34	.00	.04	.04	.34	.99	8.74	—	—
2	Adenogenic	30	2.90	42.20	3.62	11.61	90.50	10.51	.01	.10	.01	.10	.47	4.00	—	88.80
3	Odontogenic	7	2.26	30.30	3.80	10.64	80.70	8.59	.13	1.70	.02	.30	.55	7.40	—	19.37
4	Adenogenic	5	2.46	43.00	4.27	13.00	80.80	10.50	.09	1.60	.01	.20	.34	5.90	—	4.43
5	Odontogenic	22	.82	7.20	12.40	11.36	89.50	10.17	.00	.00	.00	.00	.37	3.30	—	19.52
6	Odontogenic	27	2.13	15.20	5.89	14.76	85.00	12.55	.01	.10	.00	.00	1.99	1.40	—	20.05
7	Odontogenic	14	1.95	14.80	5.20	13.17	77.00	10.14	.00	.00	.03	.20	.36	2.70	—	13.34
8	Odontogenic	15	3.26	12.30	6.54	26.49	80.50	21.32	.02	.10	.02	.10	1.14	5.80	57.92	11.45
9	Adenogenic	11	2.17	17.20	4.24	12.63	72.80	9.19	.10	.80	.09	.70	1.07	8.50	11.99	9.98
10	Odontogenic	19	.92	4.60	19.67	19.96	90.70	18.10	.04	.20	.14	.70	.76	3.80	2,193.00	12.58
11	Odontogenic	13	1.63	7.50	11.34	21.77	84.90	18.48	.00	.00	.02	.10	1.63	7.50	101.20	21.80
12	Adenogenic	10	1.29	14.50	5.14	8.90	74.50	6.63	.00	.00	.03	.30	.95	10.70	7.67	7.56
13	Adenogenic	18	1.35	9.90	7.72	12.83	81.20	10.42	.02	.10	.12	.70	1.18	7.10	87.76	7.55
14	Adenogenic	22	.34	2.20	18.68	8.68	73.20	6.35	.01	.10	.00	.00	.96	11.10	36.11	78.28
15	Odontogenic	26	.34	2.70	37.24	14.42	87.80	12.66	.00	.00	.01	.10	.46	3.70	311.50	195.93
16	Odontogenic	26	.35	2.20	41.86	15.60	93.90	14.65	.00	.00	.01	.10	.52	3.30	465.40	229.85
17	Odontogenic	21	.53	6.10	17.04	11.27	80.10	9.03	.00	.00	.02	.20	.34	3.90	27.38	66.11
18	Odontogenic	54	.33	3.80	33.82	11.71	95.30	11.16	.00	.00	.02	.20	.18	1.60	639.30	279.66

**Table 3:** Correlation between the IL-6 and CRP with lymphocyte, leukocyte, neutrophil, eosinophil, basophil, and monocyte.

	IL-6		C-reactive protein	
	*r*_s_	*P*	*r*_s_	*P*
Number of lymphocyte	-0.419	0.199	-0.517	0.017
Percentage of lymphocyte	-0.597	0.053	-0.578	0.015
Neutrophil-lymphocyte ratio	0.773	0.005	0.556	0.020
Number of leukocyte	0.518	0.102	-0.039	0.881
Percentage of neutrophil	0.927	0.001	0.515	0.035
Number of neutrophil	0.627	0.039	0.199	0.445
Number of eosinophil	-0.094	0.782	-0.560	0.020
Percentage of eosinophil	-0.110	0.747	-0.504	0.039
Number of basophil	-0.047	0.892	-0.504	0.039
Percentage of basophil	-0.075	0.826	-0.548	0.023
Number of monocyte	-0.300	0.370	-0.293	0.254
Percentage of monocyte	-0.773	0.005	-0.426	0.089

**Table 4:** Correlation between parameters and the number of the involved organs.

	Involved organs	
	*r*_s_	*P*
Number of lymphocyte	-0.376	0.124
Percentage of lymphocyte	-0.428	0.077
Neutrophil-lymphocyte ratio	0.511	0.030^∗^
Number of leukocyte	0.319	0.197
Percentage of neutrophil	0.338	0.169
Number of neutrophil	0.440	0.068
Number of eosinophil	-0.265	0.288
Percentage of eosinophil	-0.312	0.207
Number of basophil	0.108	0.670
Percentage of basophil	-0.001	0.998
Number of monocyte	-0.042	0.868
Percentage of monocyte	-0.340	0.168
IL-6	0.485	0.130
CRP	0.366	0.148

^∗^ *P* < 0.05, ^∗∗^*P* < 0.01.

**Table 5:** Correlation between number of complications and parameters.

	Number of complications		
	*r*_s_	*r*	*P* value
Number of lymphocyte	-0.342		0.165
Percentage of lymphocyte		-0.249	0.319
Neutrophil-lymphocyte ratio	—	0.576	0.012^∗^
Number of leukocyte		0.201	0.423
Percentage of neutrophils	—	0.738	0.001^∗∗^
Number of neutrophil		0.364	0.138
Number of eosinophil	-0.133		0.598
Percentage of eosinophil	-0.061		0.809
Number of basophil	0.145		0.565
Percentage of basophil	0.125		0.622
Number of monocyte		-0.070	0.738
Percentage of monocyte		-0.373	0.127
IL-6	0.907	—	0.001^∗∗^
CRP	0.599	—	0.011^∗^

^∗^ *P* < 0.05, ^∗∗^*P* < 0.01.

**Table 6:** Regression coefficients between neutrophil-lymphocyte ratio and number of the involved organs.

	Model	Nonstandardized coefficient		Standardized coefficient	*t* value	*P* value
		*β* value	Standard error			
1	Constant	4.596	0.603	—	7.617	0.001
	Neutrophil-lymphocyte ratio	0.080	0.033	0.521	2.439	0.027

**Table 7:** Regression coefficients between parameters and number of complications.

	Model	Nonstandardized coefficient		Standardized coefficient	*t* value	*P* value
		*β* value	Standard error			
1	Constant	-17.763	4.353	—	-4.081	0.003
	Percentage of neutrophils	0.257	0.052	0.855	4.937	0.001
2	Constant	-11.681	3.757	—	-3.109	0.014
	Percentage of neutrophils	0.177	0.046	0.587	3.805	0.005
	IL-6	0.002	0.001	0.463	3.004	0.017
3	Constant	-3.950	4.102	—	-0.963	0.368
	Percentage of neutrophils	0.066	0.055	0.219	1.200	0.269
	IL-6	0.002	0.001	0.551	4.525	0.003
	Neutrophil-lymphocyte ratio	0.073	0.028	0.410	2.623	0.034
4	Constant	0.949	0.397	—	2.389	0.044
	IL-6	0.002	0.001	0.634	3.152	0.001
	Neutrophil-lymphocyte ratio	0.099	0.018	0.554	5.382	0.001

## Data Availability

All the data are available upon appropriate request.
